# Diagnosing Autism Spectrum Disorder from Brain Resting-State Functional Connectivity Patterns Using a Deep Neural Network with a Novel Feature Selection Method

**DOI:** 10.3389/fnins.2017.00460

**Published:** 2017-08-21

**Authors:** Xinyu Guo, Kelli C. Dominick, Ali A. Minai, Hailong Li, Craig A. Erickson, Long J. Lu

**Affiliations:** ^1^Division of Biomedical Informatics, Cincinnati Children's Hospital Research Foundation Cincinnati, OH, United States; ^2^Department of Electrical Engineering and Computing Systems, University of Cincinnati Cincinnati, OH, United States; ^3^The Kelly O'Leary Center for Autism Spectrum Disorders, Cincinnati Children's Hospital Medical Center Cincinnati, OH, United States; ^4^School of Information Management, Wuhan University Wuhan, China; ^5^Department of Environmental Health, College of Medicine, University of Cincinnati Cincinnati, OH, United States

**Keywords:** autism spectrum disorder, resting-state fMRI, deep neural network, sparse auto-encoder, feature selection

## Abstract

The whole-brain functional connectivity (FC) pattern obtained from resting-state functional magnetic resonance imaging data are commonly applied to study neuropsychiatric conditions such as autism spectrum disorder (ASD) by using different machine learning models. Recent studies indicate that both hyper- and hypo- aberrant ASD-associated FCs were widely distributed throughout the entire brain rather than only in some specific brain regions. Deep neural networks (DNN) with multiple hidden layers have shown the ability to systematically extract lower-to-higher level information from high dimensional data across a series of neural hidden layers, significantly improving classification accuracy for such data. In this study, a DNN with a novel feature selection method (DNN-FS) is developed for the high dimensional whole-brain resting-state FC pattern classification of ASD patients vs. typical development (TD) controls. The feature selection method is able to help the DNN generate low dimensional high-quality representations of the whole-brain FC patterns by selecting features with high discriminating power from multiple trained sparse auto-encoders. For the comparison, a DNN without the feature selection method (DNN-woFS) is developed, and both of them are tested with different architectures (i.e., with different numbers of hidden layers/nodes). Results show that the best classification accuracy of **86.36%** is generated by the DNN-FS approach with 3 hidden layers and 150 hidden nodes (3/150). Remarkably, DNN-FS outperforms DNN-woFS for all architectures studied. The most significant accuracy improvement was **9.09%** with the 3/150 architecture. The method also outperforms other feature selection methods, e.g., two sample *t*-test and elastic net. In addition to improving the classification accuracy, a Fisher's score-based biomarker identification method based on the DNN is also developed, and used to identify 32 FCs related to ASD. These FCs come from or cross different pre-defined brain networks including the default-mode, cingulo-opercular, frontal-parietal, and cerebellum. Thirteen of them are statically significant between ASD and TD groups (two sample *t*-test *p* < 0.05) while 19 of them are not. The relationship between the statically significant FCs and the corresponding ASD behavior symptoms is discussed based on the literature and clinician's expert knowledge. Meanwhile, the potential reason of obtaining 19 FCs which are not statistically significant is also provided.

## Introduction

Autism spectrum disorder (ASD) is a serious lifelong condition characterized by repetitive, restricted behavior as well as deficits in communication and reciprocal social interactions (American Psychiatric Association, 2013). The traditional procedure for diagnosing ASD is largely based on narrative interactions between individuals and clinical professionals (Yahata et al., 2016). Such methods, lacking biological evidence, not only are prone to generate a high variance during the diagnosis (Mandell et al., 2007) but also require a long period to detect abnormalities (Nylander et al., 2013). As a complement to the current behavior-based diagnoses, functional magnetic resonance imaging (fMRI) has been widely applied to explore the functional characteristics or properties of a brain. It is able to assist neuroscientists to get valuable insights into different neurological disorders (Martin et al., 2016). A human brain can be understood as a complex system with various regions performing different functions. Although, some structural regions may not be connected locally, they are integrated globally to process different types of information. fMRI measuring the changes of blood oxygen level-dependent (BOLD) signal in a non-invasive way has been applied to reveal regional associations or brain networks. In 1995, Biswal et al. (1995) discovered that various brain regions still actively interact with each other while a subject was at rest (not in any cognitive task). Since then, resting-state fMRI (rs-fMRI) has become an important tool for investigating brain networks of different brain disorders such as Alzheimer's disease (Chase, 2014), schizophrenia (Lynall et al., 2010), and ASD (Monk et al., 2009) and has generated many invaluable insights into neural substrates that underlie brain disorders.

Previous ASD studies based on rs-fMRI images have examined anatomical and functional abnormalities associated with ASD from cohorts at different age levels, e.g., for the adolescence cohort (10–19 years approximately; Assaf et al., 2010; Keown et al., 2013; Starck et al., 2013; Bos et al., 2014; Chen S. et al., 2015; Doyle-Thomas et al., 2015; Iidaka, 2015; Jann et al., 2015), for the adult cohort (≥20 years) (Cherkassky et al., 2006; Monk et al., 2009; Tyszka et al., 2013; Itahashi et al., 2014, 2015; Chen C. P. et al., 2015; Jung et al., 2015), and for the cohort covered all age levels (Alaerts et al., 2015; Cerliani et al., 2015). The results helped clarify the relevant neurological foundations of ASD in different age levels. Extant literature suggests that the basic organization of functional networks is similar across all age levels. However, the levels of connectivity and modulation appear altered in ASD. The results suggested that both hypo- and hyper-connectivity occurred in ASD relative to typical developed (TD) controls. By studying an adult cohort, Monk et al. (2009) found that poorer social functioning in the ASD group was correlated with hypo-connectivity between the posterior cingulate cortex and the superior frontal gyrus, and more severe restricted and repetitive behaviors in ASD were correlated with stronger connectivity between the posterior cingulate cortex and right parahippocampal gyrus. These findings indicated that ASD adults showed altered intrinsic connectivity within the default network, and connectivity between these structures is associated with specific ASD symptoms. Assaf et al. (2010) discovered that compared to adolescent controls, adolescent ASD patients showed decreased functional connectivities (FCs) between the precuneus and medial prefrontal cortex/anterior cingulate cortex, DMN core areas, and other default mode sub-network areas. The magnitude of FCs in these regions inversely correlated with the severity of patients' social and communication deficits. Keown et al. (2013) found that local FCs were atypically increased in adolescents with ASD in temporal-occipital regions bilaterally by applying rs-fMRI and a graph model. Hyper-connectivity in the posterior brain regions was found to be associated with higher ASD symptom severity. Supekar et al. (2013) claimed that hyper-connectivity of short range connections in ASD was observed at the whole-brain and subsystems levels. It demonstrated that at earlier ages, the brains of children with ASD are largely functionally hyper-connected in ways that contribute to social dysfunction. Above finding are spread in different brain networks including cingulo-opercular (CO), default-mode (DM), cerebellum (CB), and frontal-parietal (FP).

The above findings indicated that aberrant ASD-associated FCs were widely distributed throughout the entire brain, as opposed to showing a restricted pattern within only a few specific brain regions. Thus, in this research, we developed the machine learning model to explore ASD-related FCs from the whole-brain FC pattern (FCP) which is a set of FCs including each FC between every pair of pre-defined brain regions. The method was tested on the dataset from an adolescent cohort.

Machine learning algorithms have been successfully employed in the automated classification of altered FC patterns related to ASD based on rs-fMRI images (Uddin et al., 2013). For example, Yahata et al. (2016) developed a classifier achieves high accuracy (85%) for a Japanese discovery cohort and demonstrates a remarkable degree of generalization (75% accuracy) for two independent validation cohorts in the USA and Japan. Models developed by Deshpande et al. (2013) and Nielsen et al. (2013) achieved 95.9 and 60% accuracy on a self-collected dataset and the autism brain imaging data exchange I (ABIDE I) dataset, respectively.

As an important machine learning tool, deep learning has been widely applied in various research areas (Krizhevsky et al., 2012; Graves et al., 2013). Stacking several auto-encoders (AEs) or restricted Boltzmann machine (RBMs) is a common way of building a DNN. The DNN architecture is able to improve the feature learning capacity by exploring latent or hidden low-dimensional representations which are inherent in high-dimensional raw data. With such representations, the classification model performance can be enhanced effectively. The DNN is widely used in neuroimaging studies (Brosch et al., 2013; Suk et al., 2014; Kim et al., 2016). Hjelm et al. (2014) demonstrated that the DNN constructed by stacking RBMs extracted better spatial and temporal information from fMRI data compared with ICA and PCA algorithms. Suk et al. (2015) stacked several AEs to build the DNN which was applied in a task of discriminating AD patients from mild cognitive impairment patients, and obtained the accuracy of 83.7% on the ADNI dataset. Plis et al. (2013) conducted a validation study on structural and functional neuroimaging data to show that a stacked RBMs can learn physiologically important representations and detect latent relations in neuroimaging data. Although, the DNN obtained many competitive results, its feature learning capacity is still able to be improved. Recently, researchers have tried using multiple AEs to learn better representations of the data. Zhu et al. (2014) developed a DNN-based framework to learn multi-channel deep feature representations for the face recognition task. In their design, multiple deep learning networks are applied to extract representations of different parts of the face, e.g., eyes, nose and mouth, and the combined representations used to train a classification model. Similar studies can be found in literature (Hong et al., 2015; Zhao et al., 2015). Inspired by ideas in above papers, a novel feature selection method based on multiple SAEs was proposed. Different from above studies, we constructed one SAE with high feature learning capacity by selecting features from multiple trained SAEs instead of just stacking multiple SAEs together to form several DNNs.

In this study, a DNN-based classification model has been developed for distinguishing ASD patients from TD controls based on the whole-brain FCP of each subject. The DNN model consists of the stacked SAEs for data dimension reduction, and a softmax regression (SR) on the top of the stacked SAEs for the classification. Moreover, a novel feature selection method based on multiple trained sparse auto-encoders (SAEs) has been developed. It can initialize weights between the first hidden layer and the input layer (the first feature layer) of the DNN by selecting a set of dissimilar features with high discriminative power from multiple trained SAEs. In this study, the first objective is to enhance the performance of the DNN for classifying ASD patients and TD controls by applying a novel feature selection method. The DNN-FS (3/150 hidden layers/nodes) obtained the best classification accuracy of **86.36%**. Most importantly, it outperformed the DNN-woFS for each comparison scenario. The most significant improvement, of **9.09%**, occurred when the architecture had three hidden layers with 150 nodes each. Meanwhile, by comparing the average discriminating power of the same layer in both DNN-FS and DNN-woFS, we found that the discriminating ability of any layer in DNN-FS is higher than the discriminating ability of the corresponding layer in DNN-woFS. In addition, using two other feature selection methods—a two-sample *t*-test and an elastic net—results showed that the proposed method (86.82%) outperformed both the other methods (two-sample *t*-test: 70.67%, elastic net: 79.54%). The second objective is to identify abnormal FCs related to ASD. To achieve the goal, a DNN-based biomarker identification method was developed to locate 32 FCs associated with ASD. Generally, the findings supported the points of view in literature that (1) both hyper- and hypo-connectivity exist in certain brain networks (e.g., DM) of the ASD patients; (2) abnormal between-network connectivity exists in ASD patients. More explanation is given in the Discussion section. For readers' convenience, the abbreviations of terminologies used in the following sections are listed in Table 1.

**Table 1:** Abbreviation of terminologies used in sections Materials and Methods, Results, and Discussion.

**Terminology**	**Abbreviation**
Automated anatomical labeling	AAL
Backpropogation	BP
Correlation coefficient	CC
Cross-validation	CV
Deep neural networks	DNNs
DNN with the feature selection method	DNN-FS
DNN without the feature selection method	DNN-woFS
Independent component analysis	ICA
Left crus cerebellum	LCCB
Left inferior temporal gyrus	LITG
Left pars triangularis	LPT
Left superior parietal lobule	LSPL
Mixed national institute of standards and technology	MNIST
National database for autism research	NDAR
Neural network	NN
Preprocessed connectomes project	PCP
Principal component analysis	PCA
Regions-of-interest	ROI
Right inferior temporal gyrus	RITG
Right putamen	RP
Right superior frontal gyrus	RSFG
Sparse auto-encoders	SAE
Time series	TS
University of Michigan: Sample 1	UM:S1

## Materials and methods

### Data acquisition and preprocessing

The rs-fMRI dataset used in this paper was obtained from UM:S1 site, ABIDE I (Di Martino et al., 2014). ABIDE I is the first ABIDE initiative, a grassroots consortium aggregating. The UM:S1 dataset contains samples from 110 adolescent subjects among which there are 55 ASD patients and 55 TD controls. Each sample consists of one or more rs-fMRI acquisitions and a volumetric MPRAGE image (Nielsen et al., 2013). The dataset is publicly available at http://fcon_1000.projects.nitrc.org/indi/abide/, and all the patient health information associated with the data has been de-identified. The access instructions were detailed in Di Martino et al. (2014). A diagnosis of ASD was made by applying the Autism Diagnostic Interview Revised (Lord et al., 1994) and the Autism Diagnostic Observation Schedule (Lord et al., 2000), and confirmed by clinical consensus (Lord et al., 2006). Exclusion criteria common to all participants included any history of neurological disorder (including seizures), a history of head trauma, a history of psychosis, a history of bipolar disorder, and if either verbal or non-verbal IQ score was lower than 85. The performance and verbal IQ were obtained using the Peabody Picture Vocabulary Test (Dunn et al., 1965) and the Ravens Progressive Matrices (Lord et al., 1994). Full IQ estimates were based on the average of the performance IQ and verbal IQ scores available and provided in this dataset. More details about this dataset are summarized in Table 2.

**Table 2:** Summary of demographics, neuropsychological performance, and clinical characteristics for each of the TD and ASD groups.

	**TD controls *N* = 55**	**ASD patients *N* = 55**	***t***	***p*-value**
**DEMOGRAPHICS**				
Age [Mean ± *SD* (years)]	14.2 ± 3.2	12.7 ± 2.4	2.52	0.01
Age range [youngest-oldest (years)]	8.2–19.2	8.5–18.6		
Gender (male/%)	38/69.09%	46/83.64%		
Handness (right/%)^*^	44/80%	41/74.55%		
**NEUROPSYCHOLOGICAL PERFORMANCE**				
Verbal IQ (Mean ± *SD*)	113.49 ± 13.72	107.04 ± 20.34	1.93	0.06
Performance IQ (Mean ± *SD*)^*^	100.96 ± 11.58	100.06 ± 20.08	0.28	0.78
Full IQ (Mean ± *SD*)^*^	106.85 ± 9.72	103.37 ± 17.63	1.24	0.22
**CLINICAL CHARACTERISTICS**				
ADI-R social (Mean ± *SD*)		19.76 ± 4.82		
ADI-R verbal (Mean ± *SD*)		15.43 ± 3.77		
ADOS social affect (Mean ± *SD*)		8.26 ± 3.61		

* *Missing values from some subjects were removed in calculating the mean and SD*.

Imaging was performed on a long bore 3T GE sigma scanner with a 4-channel coil at the University of Michigan's Functional MRI laboratory. For each participant, 300 ${\text{T}}_{2}^{*}$ -weighted BOLD images were collected using a reverse spiral sequence (Glover and Law, 2001). Whole brain coverage was obtained with 40 contiguous 3 mm axial slices (TR = 2,000 ms, TE = 30 ms, flip angle = 90°, FOV = 22 cm, 64 × 64 matrix). Each slice was acquired parallel to the AC-PC line. For the structural images, a high-resolution 3D T1 axial overlay (TR = 8.9, TE = 1.8, flip angle = 15°, FOV = 26 cm, slice thickness = 1.4 mm, 124 slices; matrix = 256 × 160) was acquired for anatomical localization. Additionally, a high-resolution spoiled gradient-recalled acquisition in steady state image acquired sagittally (flip angle = 15°, FOV = 26 cm, slice thickness = 1.4 mm, 110 slices) was used for registration of the functional images (Wiggins et al., 2011).

All preprocessed rs-fMRI data were obtained from the PCP which opens sharing of preprocessed neuroimaging data from ABIDE I. PCP applied four pipelines to preprocess the rs-fMRI data. Processing steps in these pipelines are highly similar. However, the algorithm implementation and parameters used in each step among different pipelines are specific. Although, there is no consensus on the best method for preprocessing rs-fMRI data, it is generally accepted that “scrubbing” the data of motion artifact outliers provides a certain level of protection against this motion-induced bias. The subject's motion produces substantial changes in the timecourses of rs-fMRI data, and can cause systematic but spurious correlation structures throughout the brain. Specifically, many long-distance correlations are decreased by subject motion, whereas many short-distance correlations are increased (Power et al., 2012; Satterthwaite et al., 2013; Yan et al., 2013). These artifacts can distort the strength of FCs, and further affect the diagnosis of all kinds of neurological disorders such as ADHD and ASD (Fair et al., 2012; Alaerts et al., 2015). Among the four pipelines, NIAK includes the “volume scrubbing” step for reducing the head motion effect. In NIAK, the raw rs-fMRI data were preprocessed using the following steps: motion realignment, intensity normalization (non-uniformity correction using median volume), nuisance signal removal, and registration. In the nuisance signal removal step, the scrubbing was employed to clean confounding variation due to physiological processes (heart beat, head motion, and respiration), and head motion. The low frequency scanner drifts from the fMRI signal were cleaned by setting up a discrete cosine basis with a 0.01 Hz high-pass cut-off. The band-pass filtering (0.01–0.1 Hz) was applied after nuisance variable regression. In the registration step, the data were spatial normalized to the Montreal Neurological Institute template with 3 mm isotropic voxel size, followed by spatial smoothing using a 6-mm isotropic full-with at half maximum Gaussian kernel. For more data preprocessing details, please refer to Di Martino et al.'s paper (Di Martino et al., 2014). The description of pipelines can be found at http://preprocessed-connectomes-project.org/abide/Pipelines.html.

**Figure 1:**
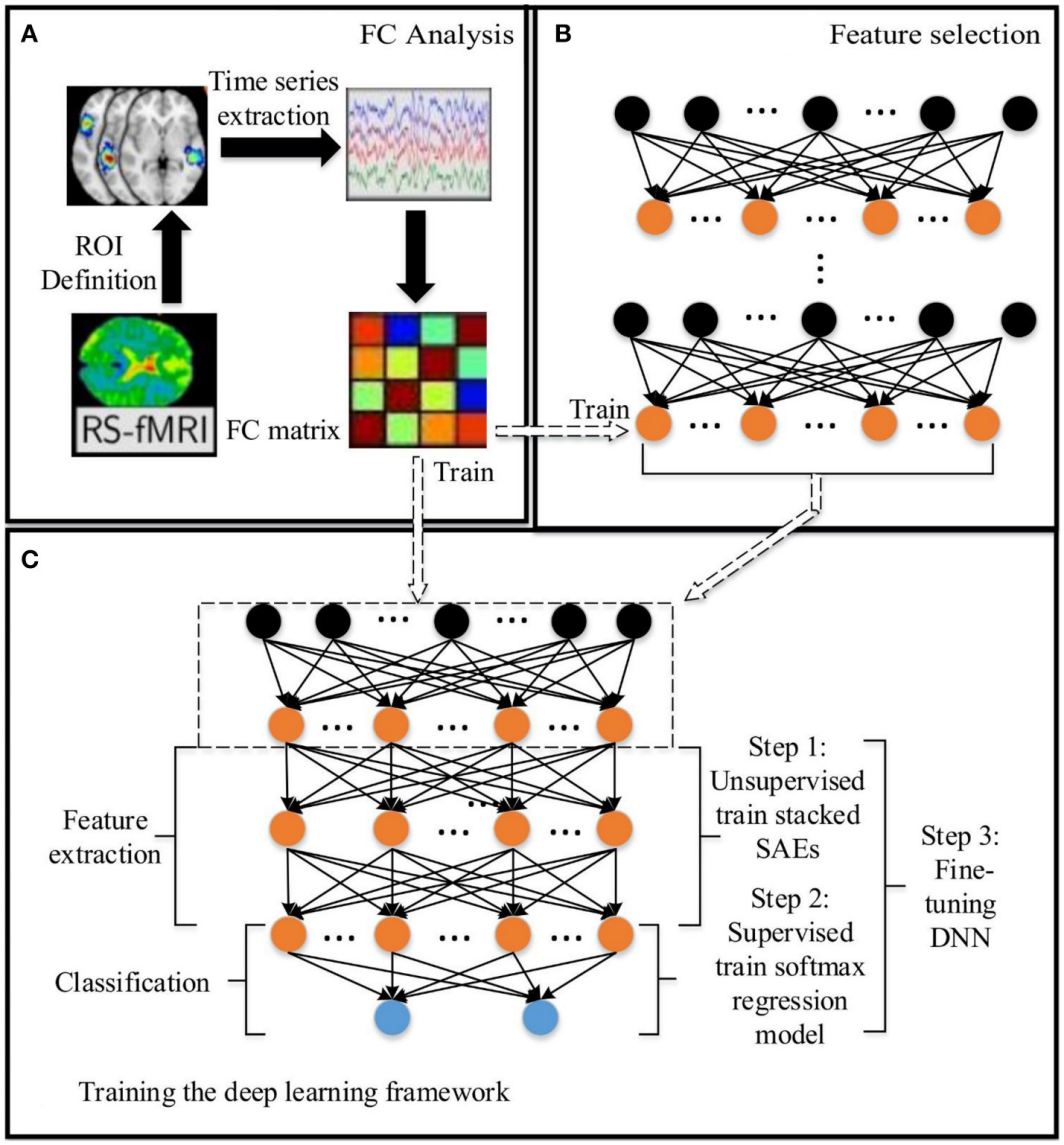
The DNN based method for predicting the ASD: **(A)** Functional connectivity (FC) analysis. **(B)** Feature selection based on SAEs. **(C)** Training the DNN. The dashed arrow between **(C)** and **(B)** indicates that the low level SAE in **(C)** comes from **(B)**. Arrows between **(A)** and **(B)**, **(A)** and **(C)** indicate the data flow between modules.

Neuroscientists at PCP extracted mean time-series for several sets of ROIs atlases, including AAL which can be obtained at http://preprocessed-connectomes-project.org/abide/Pipelines.html. The mean BOLD TS across voxels in each region of AAL was calculated from each rs-fMRI already registered in standard space. This project used mean BOLD TS from the AAL atlas containing 116 regions. Then Pearson's CCs were calculated using the resulting mean BOLD TS from all 6670 ($C_{116}^{2}$) possible pairs of regions. Figure 1A illustrates all steps of getting the whole-brain FCP. However, with the help of PCP, only the final step was needed, i.e., calculating the functional connectivity matrix based on BOLD TS of each voxel in each pair of regions. As with any statistic, Pearson's CCs has a sampling distribution. It will be normally distributed when the absolute value of the correlation in the population is low. However, if high correlation values occur frequently in the population, the distribution has a negative skew effect. To avoid this and force samples to be normally distributed, CCs were Fisher's r-to-z transformed (Rosner, 2015). Then z-scores of each subject were normalized (mean = 0, standard deviation = 1) via pseudo z-scoring. Thus, each subject's measurements were represented as a vector of 6,670 z-scores—one for each pair of the 116 brain regions. This vector is designated as the FCP for that subject, and is used as input to the classifiers. Each element of the FCP vector is termed a FC.

### Overview of the method

In this paper, a DNN model with a novel feature selection method based on multiple SAEs was proposed for predicting ASD from brain resting-state functional connectivity patterns. The entire model contains three parts: (A) functional connectivity analysis, (B) feature selection based on multiple SAEs, and (C) training the DNN (Figure 1). First, each raw rs-fMRI data was preprocessed, and the whole-brain FCP were obtained by calculating the Pearson's CC of TSs from any pair of ROIs. Let *X* ∈ *R*^*N*×*d*^ denote the training data, in which each row indicates a training sample, and *Y* ∈ *R*^*N*^ denote their corresponding labels. As discussed above, the dimension of each whole-brain FCP is 6,670 (*d* = 6670). Multiple SAEs were then trained on the training data using gradient descent learning. The hidden neurons of these SAEs after training provided a feature pool. The feature selection algorithm was then used to select features with high discriminating power, which were used to form the first hidden layer of the DNN model (marked by the dashed rectangular box in Figure 1C). It is hypothesized that this layer provides low-dimension representations with higher quality compared to representations obtained from a single trained SAE, and such representations would allow the DNN to improve the classification accuracy. Next, several SAEs and an SR model were stacked on top of the constructed feature layer to form a DNN, which was trained (excluding the first hidden layer) by following steps shown in Figure 1C using labeled training data.

**Figure 2:**
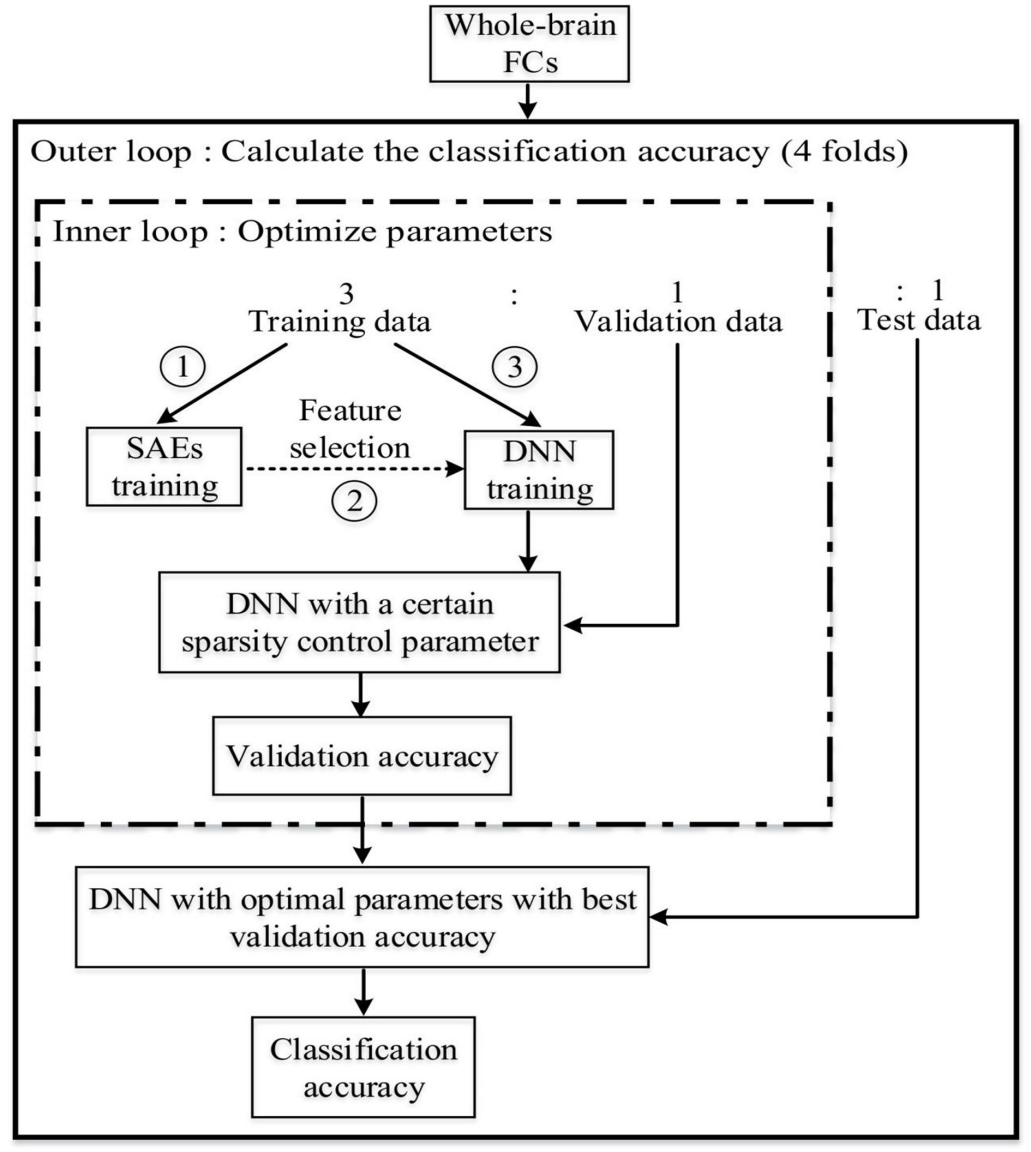
The nested CV framework for the DNN training, testing, and parameters optimization.

For training and testing the DNN, and optimizing parameters in the model, the five-fold nested CV framework shown in Figure 2 was used. After functional connectivity analysis, the obtained whole-brain FCPs were applied as input to the DNN. The DNN classifier was trained and parameters were optimized using training and validation data during the training phase. Then the classification task was performed on each individual in the test data in the test phase to identify the corresponding status (ASD patient or TD control). Finally, the weights of the DNN were analyzed to understand patterns learned by it and to determine ASD-related FCs.

### The novel feature selection method based on multiple sparse auto-encoders

#### Sparse auto-encoders

**Figure 3:**
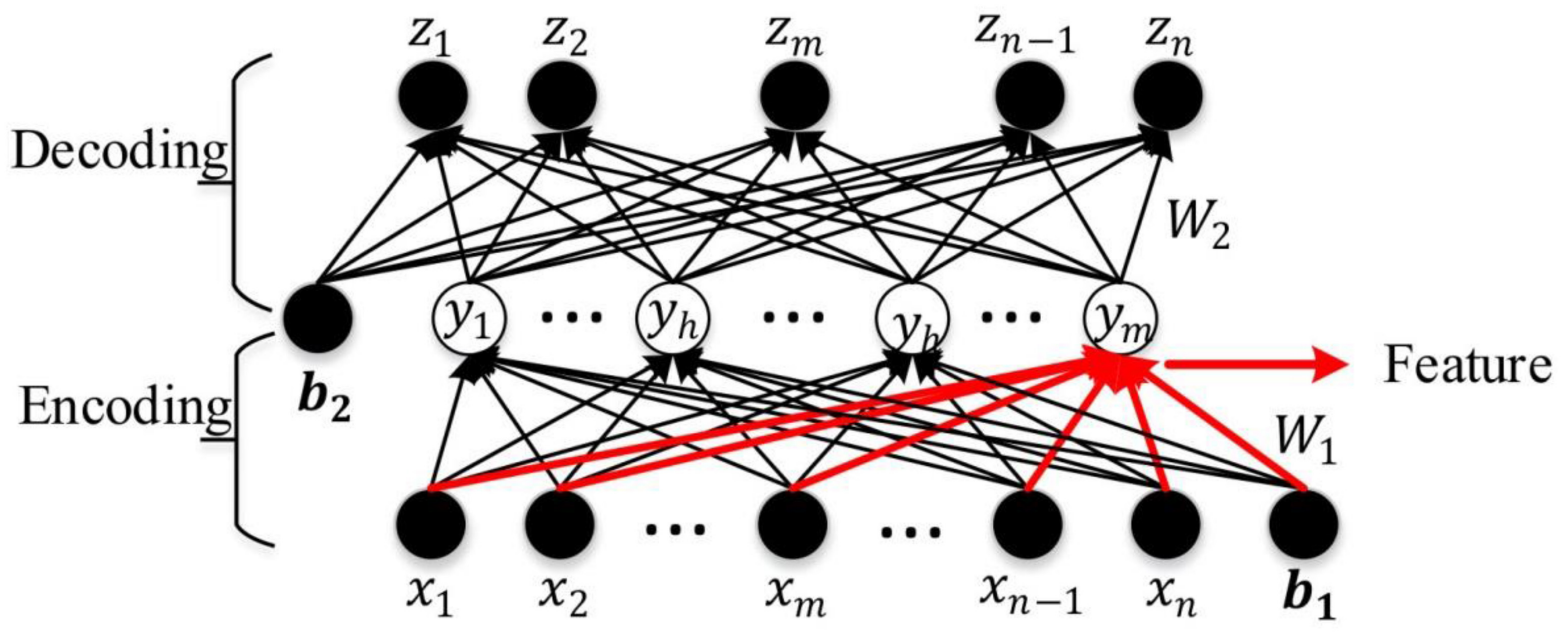
The schematic structure of the SAE.

An AE is a three-layer feed-forward NN as shown in Figure 3. It comprises an input layer, a hidden layer, and an output layer. The hidden layer is fully connected to the input layer and the output layer through weighted feed-forward connections. The AE is trained so that its output layer reproduces the stimulus pattern on its input layer. Thus, the number of nodes in the input layer and the output layer is the same, and are both equal to the dimension of the data. Typically, the number of hidden nodes in an AE is much smaller than the number of input and output nodes. To achieve the required reconstruction of its input at the output layer, the AE is forced to infer a maximally information-preserving lower-dimension representation of the input in the hidden layer, which can then be mapped to the output layer. Thus, such an AE performs a dimension reduction function, and each hidden node can be seen as representing a feature of this lower-dimension representation. A convenient way to visualize the feature represented by a hidden node is the pattern of its connection weights from all input nodes (marked in red in Figure 3). The AE is called an SAE if a sparsity constraint is imposed on the mean activity of the hidden layer nodes (Larochelle et al., 2009) to reduce overfitting. The number of nodes in the input and output layers is denoted by *d* (each variable of the observation maps to a node) and the number in the hidden layer by *m* (*m* ≤ *d*); *W*^(1, 0)^ ∈ *R*^(*m*×*d*)^ and *W*^(2, 1)^ ∈ *R*^(*d*×*m*)^, respectively, denote the encoding weight matrix and the decoding weight matrix; *b*^(1, 0)^ ∈ *R*^*m*^ and *b*^(2, 1)^ ∈ *R*^*d*^ are, respectively, the bias vectors for the hidden layer and the output layer. The SAE learns a compressed representation of its input by minimizing the reconstruction error between its input pattern and the reconstructed output pattern (Suk et al., 2015).

Let *x* = [*x*_1_, *x*_2_, …*x*_*w*_, …*x*_*d*−1_, *x*_*d*_] be an observation from the training data set, *y* = [*y*_1_, *y*_2_, …, *y*_*h*_, …*y*_*n*−1_, *y*_*m*_] the compressed hidden layer representation of ***x*** (*y*_*h*_ denotes any hidden layer node between *y*_1_ and *y*_*m*_), and *z* = [*z*_1_, *z*_2_, …, *z*_*w*_, …*z*_*d*−1_, *z*_*d*_] the corresponding output vector. The mapping from ***x*** to ***y*** is given by *y* = *f*(*W*^(1, 0)^*x* + *b*^(1, 0)^), where *f* ( ) is the non-linear activation function of the hidden layer nodes. The work reported in this paper uses a logistic sigmoid function (Equation 1) which is widely used in machine learning and pattern recognition tasks (Bengio et al., 2007; Lee et al., 2007; Ngiam et al., 2011; Shin et al., 2013).

(1) f(u)=1/(1+exp(−u))

The compressed representation ***y*** in the hidden layer is mapped to the output ***z*** by a similar function:

(2) $$z=f\left(W^{\left(2,1\right)}y+b^{\left(2,1\right)}\right)$$

The reconstruction error at the output layer is measured as:

(3) $$J\left(W,b;x^{\left(i\right)},z^{\left(i\right)}\right)=\frac{1}{2}||z^{\left(i\right)}-x^{\left(i\right)}|{|}_{2}^{2}$$

where *x*^(*i*)^ is the *i*th input pattern from the training dataset and *z*^(*i*)^ is the corresponding output of the network for the given input. The network is trained by minimizing a two-term cost function given by:

(4) $$Cost=J\left(W,b\right)+\beta \sum_{j=1}^{m}KL(\rho ||\rho \prime j)$$

The first term *J*(*W, b*) also has two parts:

(5) $$J(W,b)=\frac{1}{M}\sum_{i=1}^{M}J(W,b;x^{\left(i\right)},y^{\left(i\right)})+\frac{\lambda }{2}\sum_{l=0}^{n_{l}-2}\sum_{i=1}^{s_{l+1}}\sum_{j=1}^{s_{l}}{\left(W_{ij}^{\left(l+1,l\right)}\right)}^{2}$$

where *M* denotes the total number of observations in the training dataset; *n*_*l*_ indicates the number of layers in the network (*n*_*l*_ = 3 here); *s*_*l*_ denotes the number of nodes in layer *l*; and $W_{ij}^{\left(l+1,l\right)}$ is the weight of the connection from node *i* in layer *l*+*1* to unit *j* in layer *l*. The first term represents the reconstruction error averaged over the training set, while the second term—called weight decay—is a regularization term that tends to decrease the total magnitude of the weights in the SAE and helps prevent overfitting. The parameter λ controls the relative importance of the two terms.

The second term in the *Cost* function is also a regularization term called a sparseness constraint. Its purpose is to ensure that only a small number of hidden nodes have significant activity for any given input pattern. Let *y*_*j*_(*x*) denote the activation of the hidden node *j* for input ***x***. Then the average activation of the hidden node *h* over the entire training dataset is given by:

(6) $$\rho \prime_{j}=\;\frac{1}{M}\;\underset{i=1}{\sum^{M}}\left[y_{j}\left(x^{\left(i\right)}\right)\right]$$

A target sparsity parameter ρ is defined as a small value (e.g., 0.05) such that ρ′ *j* is required to be close to ρ for all hidden units *j*. This means that any individual hidden node must be inactive for most inputs (e.g., active only in about 5% of cases if ρ = 0.05). This is accomplished by adding the second term to the *Cost* function that penalizes ρ′ *j* deviating significantly from ρ. Since ρ and ρ′ *j* can be seen as the probabilities of Bernoulli random variables to be 1, a Kullback-Leibler (KL) divergence (Shin et al., 2013), denoted by the following formula, is used as the penalty term:

(7) $$\;KL(\rho ||\rho \prime_{j})=\rho \;log\frac{\rho }{\rho \prime_{j}}+\left(1-\rho \right)log\frac{1-\rho }{1-\rho \prime_{j}}$$

This has the characteristic that *KL*(ρ||ρ′*j*) = 0 if ρ′*j* = ρ, and the value increases as ρ′ *j* deviates from ρ. The optimization of the SAE using the *Cost* function in Equation (4) ensures that the average reconstruction error for training data and the deviation of hidden node activity from the target sparsity value are both minimized, with β as the parameter controlling the relative significance of the two objectives. A standard neural network training method called BP (Werbos, 1974) was used in conjunction with an optimization method called limited-memory Broydon-Fletcher-Goldfarb-Shanno optimization (L-BFGS; Liu and Nocedal, 1989) to obtain optimal parameters of the SAE. The parameters of the SAEs, including the target sparsity ρ, and the objective weighting parameters λ and β were set using typical values recommended by other researchers: ρ = 10^−2^, λ = 10^−4^. Each SAE was trained until the *Cost* function converged.

#### The novel feature selection method

As described above, each set of connection weights from the input layer to a specific hidden node define a feature of the trained SAE. The activation of the *ith* (0 < *i* ≤ *m*) hidden layer node is given by Equation (8), where $W_{ij}^{\left(1,0\right)}$ denotes the connection weight from node *j* in the input layer to node *i* in the hidden layer, *p*_*j*_ denotes the *jth* component in the whole-brain FCP, ${b_{i}}^{\left(1,0\right)}$ denotes the bias for hidden node *i*, and *f* is the logistic sigmoid function:

(8) $$a_{i}=f(\sum_{j=1}^{n}W_{ij}^{\left(1,0\right)}p_{j}+{b_{i}}^{\left(1,0\right)})$$

This can be seen as a filter tuned to a particular input pattern ***x*** which maximizes *a*_*i*_. This pattern can be specified uniquely by constraining the norm of the input whole-brain FCPs as:

(9) $$||x|{|}^{2}=\sum_{i=1}^{n}p_{i}^{2}\leq 1$$

Then the input pattern that maximally activates hidden node *i* is given by setting the FC *p*_*j*_ (for all *n* FCs, *j* = 1,…,*n*) to:

(10) $$p_{j}=\frac{W_{ij}^{\left(1,0\right)}}{\sqrt{\sum_{j=1}^{n}{\left(W_{ij}^{\left(1,0\right)}\right)}^{2}}}$$

which defines the pattern of whole-brain FCP which evokes a maximal response from hidden node *a*_*i*_ and is, therefore, the feature defined by that node.

**Figure 4:**
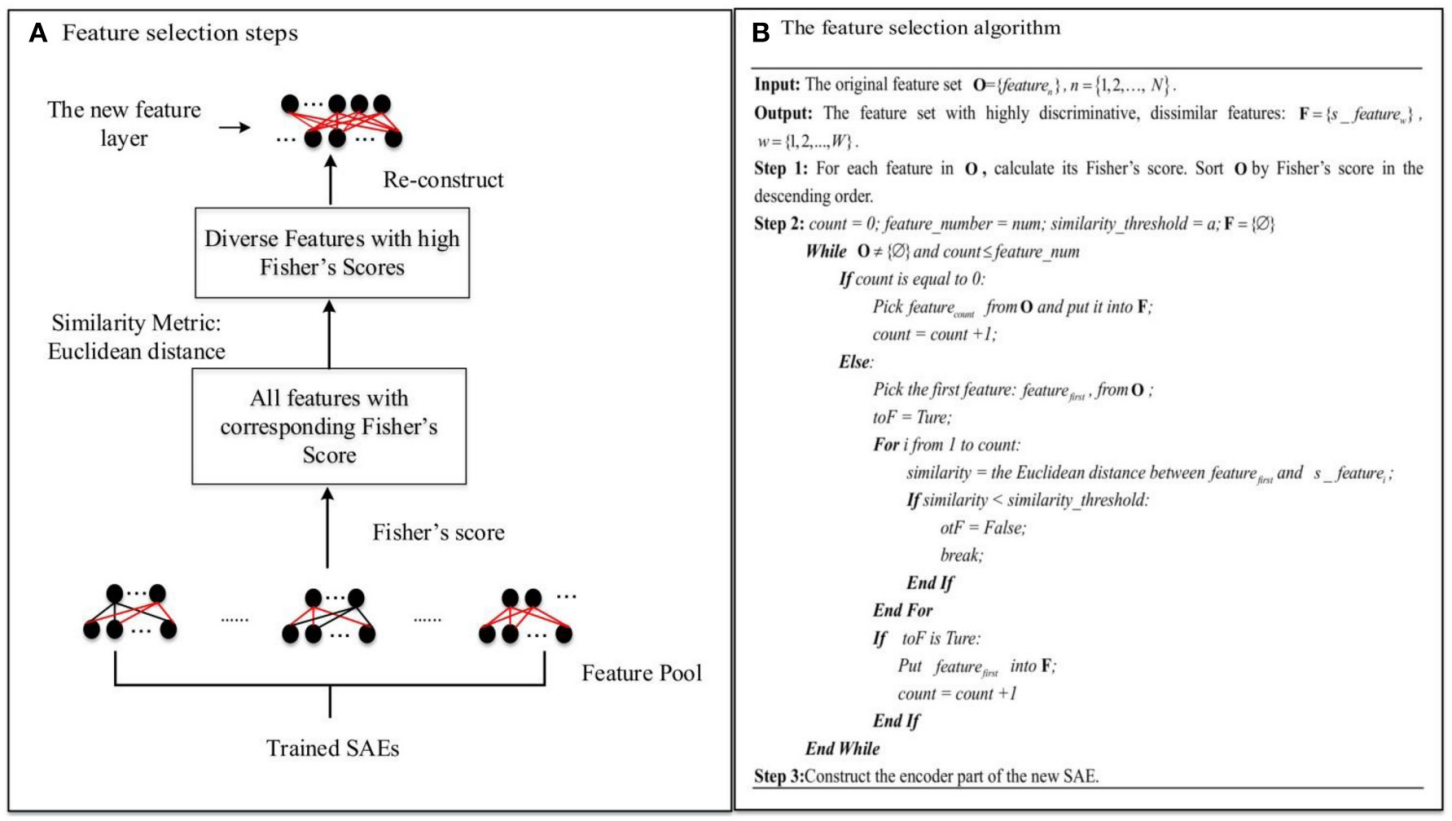
The novel feature selection method: **(A)** Feature selection steps. **(B)** The feature selection algorithm.

Many features of the whole-brain FCP can be detected by a single trained SAE, e.g., an SAE with 200 hidden layer nodes can learn 200 features of whole-brain FCP, each of which fires the corresponding node maximally. However, redundant features often emerge in the feature layer, so that many hidden layer nodes have very similar activation values, reducing the representational range of the hidden layer. Typically, even with regularization, the number of diverse features in a single trained SAE after excluding redundant features is not enough for a sufficiently informative compressed representation. The proposed novel feature selection method based on multiple SAEs can address this problem by selecting a number of diverse features with high discriminating power (ASD vs. TD) from a large diverse but redundant feature pool. The accuracy of the classification model trained by such representations from the new feature layer should improve as a result. Figure 4 illustrates the steps of the proposed feature selection method and the associated algorithm in detail.

As Figure 4A illustrates, first of all, *L* (*L* = 15 in this study) SAEs, each with *m* hidden layer nodes, are trained on the three-fold training dataset as shown in Figure 2. *L*—the number of SAEs used to generate the feature pool—is an important parameter. Using a larger value of *L* results in a larger, but possibly more redundant, feature pool. Using a smaller L produces a smaller feature pool with less feature diversity, which would cause degraded performance. In our study, we actually tried different values of *L* (5, 10, 15, 20) to decide the appropriate number, and it turned out that the deep learning framework associated with the proposed feature selection method obtained the best result in the ASD vs. TD classification task when *L* is equal to 15. The *L*×*m* features collected from these form a diverse, redundant feature pool. The next step of the feature selection algorithm is to determine the discriminating power of each feature in the pool by calculating its Fisher's score (Weston et al., 2000), which can be defined as Equation (11):

(11) $$F_{i}=\left|\frac{{\left(E_{i}^{TD}-E_{i}^{ASD}\right)}^{2}}{{\left(\sigma_{i}^{TD}\right)}^{2}-{\left(\sigma_{i}^{ASD}\right)}^{2}}\right|$$

Here, $E_{i}^{TD}$ is the mean activation value of the *i*th hidden layer node for all inputs in the TD group, i.e.,

(12) $$E_{i}^{TD}=\frac{1}{N^{TD}}\sum_{k=1}^{N^{TD}}a_{i}^{k}=\frac{1}{N^{TD}}\sum_{k=1}^{N^{TD}}\sum_{j=1}^{d}W_{\left(i,j\right)}^{\left(1,0\right)}sgm(x_{j}^{\left(k\right)})$$

where *sgm*() is the sigmoid function; $\sigma_{i}^{TD}=\frac{1}{N^{TD}}\sum_{k=1}^{N^{TD}}{\left(a_{i}^{k}-E_{i}^{TD}\right)}^{2}$ is the variance of the hidden node input for the TD group; *N*^*TD*^ and *N*^*ASD*^ are the number of subjects in the TD control and ASD groups, respectively; and $W_{\left(i,j\right)}^{\left(1,0\right)}$ is the connection weight to the *i*th node in the hidden layer from the *j*th node in the input layer. $E_{i}^{ASD}$ and $\sigma_{i}^{ASD}$ are defined similarly. The feature is considered to have high discriminating power (ASD vs. TD) when its Fisher's score is high. Features with high discriminating power are of interest during the feature selection procedure due to the fact that they can generate more informative representations for the classification model. For selection, features in the pool are sorted by their Fisher's scores in descending order. Then a number of dissimilar features from the pool with high discriminating power are selected. In order to choose diverse features, a Euclidean distance similarity metric is applied to measure the similarity of a pair of features. Figure 4B illustrates the procedure for feature selection in detail. *feature*_*number* indicates the number of features to be selected from the pool, and *similarity*_*threshold* indicates a threshold for the feature pair similarity. The algorithm considers features in descending order of discriminating power. The criterion for selection is that a feature should be selected only if its similarity to any other features in the set **F** of already selected features is below the *similarity_threshold* value. Otherwise, the next feature is considered. This procedure is not terminated until the number of selected features is equal to *feature*_*number*, or all features in the pool have been examined. Finally, a diverse and highly discriminating feature layer is re-constructed using features collected in **F**.

#### Training the DNN model

**Figure 5:**
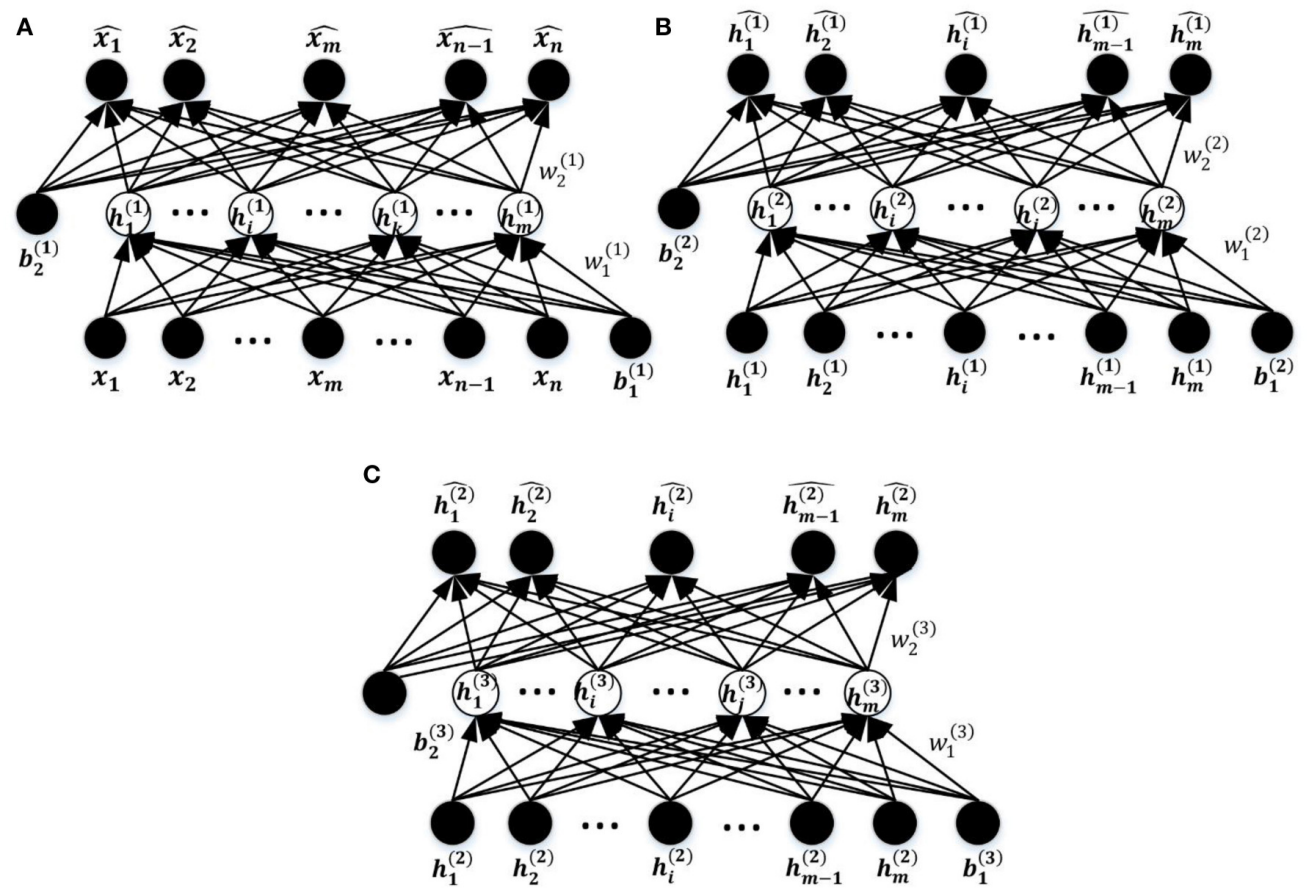
The process of layer-wise training the stacked SAEs. **(A)** The first SAE. **(B)** The second SAE. **(C)** The Third SAE.

The entire DNN training process comprises three steps as illustrated in Figure 1C, including unsupervised training of the stacked SAEs, supervised training of the SR model, and fine tuning of the entire DNN. The first two steps are considered to be pre-training of the DNN model. The DNN is a multilayer NN constructed by stacking multiple SAEs and the SR model together. The stacked SAEs are used to do successive data dimension reductions, and the final reduced-dimension representation is provided as input to the SR model. In the DNN, weights in lower layers are difficult to update by the standard back-propagation algorithm. This is because the algorithm involves the backward propagation of objective function gradients from upper layers to lower layers, and these vanish quickly as the depth of the network increases (Hinton, 2007). As a result, weights in lower layers change slowly, causing the lower layers to not learn much. A layer-wise pre-training method (Hinton et al., 2006) can greatly improve the training process of a multi-layer NN, and is widely used in various DNN-based applications (Suk et al., 2014, 2015; Kim et al., 2016). This is the approach used to train the current model. Figure 5 shows the three SAEs used to form the stacked SAEs and illustrates the layer-wise training process. The lowest SAE was first trained with the training dataset (Figure 5A). Then each image *x* was used as input to the trained SAE to get the corresponding hidden layer activation vectors *h*^(1)^ that are considered to represent latent features of the data. After collecting this representation for each image, the middle SAE was trained using these collected representations as input as desired output, as Figure 5B illustrates. Then the hidden layer activation vectors *h*^(2)^ for each data point were collected from this SAE and used as inputs to train the next SAE. This process is shown in Figure 5C. Finally, the outputs of the third SAE's hidden layer, *h*^(3)^, that are considered as representing the most complex non-linear features latent in the raw data, were generated in the same way as *h*^(1)^ and *h*^(2)^. The output of *h*^(3)^ was then used as input to train the SR model. The data label information was not involved in the entire layer-wise training process of the stacked SAEs, so it was considered to be unsupervised training. The one exception is the training of the lowest level SAE, which uses the feature selection algorithm and uses labeled data to calculate the discrimination power of features. The SR model is trained by both training data representations and the corresponding labels so the training process is supervised. The pre-training is able to provide a reasonable initialization of parameters for the fine-tuning step (Erhan et al., 2010) so that parameters can be adjusted quickly according to training data labels in a few training iterations.

The ultimate goal of training the DNN is to construct a diagnosis model that distinguishes ASD patients from TD controls. In the fine-tuning step, all training data associated with labels were used to train the DNN. The BP algorithm in conjunction with L-BFGS was applied to optimize all the weights. The cost function of the DNN is defined by Equation (13) and Equation (14).

(13) $$Cost=\frac{1}{M}\sum_{k\;=\;1}^{M}J(W,b;x^{\left(k\right)},y^{\left(k\right)})\;+\;\frac{\lambda }{2}\sum_{l\;=\;0}^{n_{l}-2}\sum_{i\;=\;1}^{s_{l\;+\;1}}\sum_{j=1}^{s_{l}}{\left(W_{ij}^{\left(l\;+\;1,l\right)}\right)}^{2}$$

(14) $$J\left(W,b;x^{\left(k\right)},y^{\left(k\right)}\right)=\frac{1}{2}||h_{W,b}(x^{\left(k\right)})-y^{\left(k\right)}|{|}_{2}^{2}$$

In Equation (13), *M* denotes the total number of subjects in the training dataset, *W* denotes the weights parameters in the DNNs, *b* denotes the bias vector, *x*^(*k*)^ denotes the input for *k*th subject in the training dataset, *y*^(*k*)^ is its corresponding label which can indicate the condition status (ASD 1, TD control 0), *n*_*l*_ indicates the total number of layers of the DNNs, *s*_*l*_ indicates the total number of nodes in the layer *l*, $W_{ij}^{\left(l+1,l\right)}$ presents the weight between *i*th node in layer *l*+1 and the *j*th node in the layer *l*. From Equation (4), it can be seen that *J*(*W, b*; *x*^(*i*)^, *z*^(*i*)^) measures the error between the predicted label of a certain subject $h_{W,b}\left(x^{\left(k\right)}\right)$ and the corresponding real label *y*^(*k*)^. Based on the cost function, the weights optimization process can be described as following::

(15) $$\begin{array}{l} {W_{ij}}^{\left(l+1,l\right)}(t+1)={W_{ij}}^{\left(l+1,l\right)}(t)-\alpha (t)[(\frac{1}{M}\delta {W_{ij}}^{\left(l+1,l\right)}(t))\\ \;+\;\lambda {W_{ij}}^{\left(l+1,l\right)}(t)] \end{array}$$

(16) $${b_{j}}^{\left(l+1,l\right)}(t+1)={b_{j}}^{\left(l+1,l\right)}(t)-\alpha (t)[\frac{1}{M}\delta {b_{j}}^{\left(l+1,l\right)}(t)]$$

(17) $$\delta {W_{ij}}^{\left(l+1,l\right)}(t)=\sum_{k=1}^{M}\frac{\partial }{\partial W_{ij}^{\left(l+1,l\right)}(t)}J(W(t),b(t);x^{\left(k\right)},y^{\left(k\right)})$$

(18) $$\delta {b_{j}}^{\left(l+1,l\right)}(t)=\sum_{k=1}^{M}\frac{\partial }{\partial b_{j}^{\left(l+1,l\right)}(t)}J(W(t),b(t);x^{\left(k\right)},y^{\left(k\right)})$$

where δ*W*^(*l*+1, *l*)^(*t*) is the sum of partial derivatives of the cost function with respect to *W*^(*l*+1, *l*)^(*t*); δ*b*^(*l*+1, *l*)^(*t*) is the sum of partial derivatives of the cost function with respect to $b_{j}^{\left(l+1,l\right)}\left(t\right)$, and *t* is the current iteration number. In this work, all of the training data was used to update parameters during each epoch until the cost function converged. The weight decay parameter λ was fixed to 10^−5^ to prevent overfitting of weights (Moody et al., 1995), the learning rate was initially set to 0.0015 and then gradually reduced after 200 epochs (Darken and Moody, 1990). The number of training epochs was 400.

#### The learnt DNN features

FCPs from all 110 subjects were used to train the DNNs once rather than using the nested CV scheme illustrated in Figure 2. This training scheme can save the complication of merging DNN weights obtained from different training sets although the classification accuracy is not available simultaneously. The sparsity parameter was set to the value selected most frequently for the final test during the nested CV scheme.

Analyzing the weights from the input layer to hidden nodes and between successive layers of hidden nodes can help in explaining the basis of classification learned by DNNs. Features in DNNs trained on the MNIST dataset (Haykin and Kosko, 2001) have been shown to correspond to simple image components such as edges by analyzing the weights between the input and the first hidden layers, and the linear combination of these features to the second layer can be seen as detecting more complex elements such as corners. An approach similar to these and other studies (Denil et al., 2013; Suk et al., 2014; Kim et al., 2016) was used to analyze the feature for each hidden layer node in each hidden layer in the DNNs. The feature vector of *i*th node in the (*l* + 1)th layer is specified as:

(19) $$F_{i}^{l+1}=\sum_{j=1}^{Z}W_{\left(i,j\right)}^{\left(l+1,l\right)}F_{j}^{l}\text{ and }F_{i}^{1}=W_{\left(i,:\right)}^{\left(1,0\right)}$$

**Figure 6:**
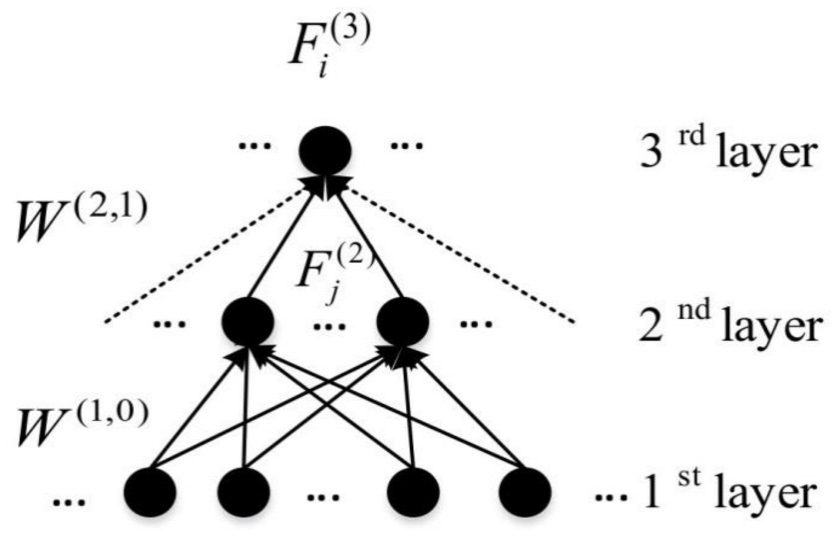
An example of the representation of learnt features from DNN (Connections used to construct features are presented by solid arrows).

Here, $W_{\left(i,j\right)}^{\left(l+1,l\right)}$ is the weight between the *i*th node in the (*l* + 1)th layer and the *j*th node in the layer *l*. To define the feature for each hidden node, the *Z* connections with the highest weight magnitudes between (*l* + 1)th and *l*th layer were chosen, and used in the analysis of features. For each node in the first hidden layer, the weights between itself and all input nodes are considered as the learnt feature. So $W_{\left(i,:\right)}^{\left(1,0\right)}$ which is actually $F_{i}^{1}$, indicates all weights of connections connecting *i*th node in the first hidden layer to all nodes from the input layer. Figure 6 illustrates the feature construction procedure for a hidden layer node in the third layer. It can be seen that a feature existing in the higher hidden layer of the DNN is actually the linear combination of features from lower hidden layers. Because the DNN features have the hierarchical property, a top-down ASD related biomarker identification method can be developed. The detailed procedure and the result will be detailed in Section Visualization of Significant FCs Identified by Learning.

## Results

### Comparing the performance of the DNN with and without the feature selection method

To assess the benefit of the proposed feature selection method, the performance of the DNN-FS illustrated in Figure 1 was compared with that of the DNN-woFS. In DNN-FS, the weights between the first hidden layer and the input layer of the stacked SAEs were selected by the proposed feature selection method, and were trained further during the 3-step DNN training. All other weights were initialized randomly. The training proceeded from the second hidden layer onward, i.e., weights to the second hidden layer from the first were trained first, then those from the second to the third, and so on). The reduced-dimension representations of the whole-brain FCPs from the first hidden layer were considered as the input to the network from the second hidden layer on. In DNN-woFS, all weights were randomly initialized and training proceeded from the first hidden layer with the whole-brain FCPs were the input. The hypothesis was that the stacked SAEs in DNN-FS would generate reduced-dimension, highly discriminative representations with higher quality than those generated by DNN-woFS, and that, after training, this would be visible in the classification accuracy of the SR classifiers using each system. Several comparison scenarios were designed to test the hypothesis. In each scenario, DNN-FS and DNN-woFS had the same configuration in the number of hidden layers and the number of hidden layer nodes. This configuration varied among different scenarios so that the performance of the systems could be evaluated with different architectures. The evaluation scheme for both models in each scenario was the five-fold CV, and both used the parameter settings discussed in Section Sparse Auto-Encoders and The Novel Feature Selection Method.

Tables 3, 4 present the classification accuracy from DNN-FS and DNN-woFS, respectively in different scenarios. From Table 3 it is clear that, for the DNN-woFS, the best performance was 81.82% when it had three hidden layers, each of which had 100 nodes. The best performance in Table 4 was 86.36% coming from the DNN-FS with three layers, each of which had 150 hidden nodes. From both tables, it can also be observed that simply increasing the number of hidden layer nodes does not always help improve accuracy when the number of hidden layers stays the same. Similarly, simply increasing the number of layers does not always help the stacked SAEs generate more robust representations when the number of nodes is fixed.

**Table 3:** Results from the DNN-woFS in different scenarios.

**No. of nodes**	**No. of Layers**				
	**1 (%)**	**2 (%)**	**3 (%)**	**4 (%)**	**5 (%)**
50	56.36	61.82	70.91	66.36	67.27
100	63.64	71.82	**81.82**	77.27	74.55
150	65.45	70.00	77.27	73.63	70.91
200	68.18	70.91	76.36	78.18	76.36

*The best performance is marked with bold*.

**Table 4:** Results from the DNN-FS in different scenarios.

**No. of nodes**	**No. of Layers**				
	**1 (%)**	**2 (%)**	**3 (%)**	**4 (%)**	**5 (%)**
50	59.09	64.55	72.73	67.27	69.09
100	67.27	75.45	85.45	81.82	77.27
150	68.18	78.18	**86.36**	75.45	72.73
200	69.09	72.73	77.27	79.09	78.18

*The best performance is marked with bold*.

The results in Tables 3, 4 provide strong support for the hypothesis that the feature selection method used in DNN-FS networks helps the stacked SAEs generate more competitive representations for the ASD diagnosis task. Compared with the results from DNN-woFS, DNN-FS classification accuracy was always higher in every comparison scenario (Table 5). The color scale green-yellow-red corresponds to low-medium-high improvement, while the numerical values indicate the exact percentage improvement. The most significant improvement (9.09%) occurs in the DNN-FS with three hidden layers, each of which has 150 nodes. However, the improvement of the DNN-FS is low when each hidden layer contains 200 nodes, even when the number of hidden layers is varied.

**Table 5:** The improvement of the DNN-FS in different scenarios compared with the DNN-woFS.

**No. of nodes**	**No. of layers**				
	**1 (%)**	**2 (%)**	**3 (%)**	**4 (%)**	**5 (%)**
50	2.73	2.73	1.82	0.91	1.82
100	3.63	3.63	3.63	4.55	2.72
150	2.73	8.18	9.09	1.82	1.82
200	0.91	1.82	0.91	0.91	1.82

### Analysis of layer-wise discriminative power

To quantitatively determine the discriminative power of each hidden layer in the DNN-FS and the DNN-woFS, DNN-FS and DNN-woFS networks with the same architecture were trained on FCPs from all subjects, and the discriminative powers of the corresponding layers were determined by calculating the mean Fisher's score and its standard deviation for each layer. Since the DNN-FS with three hidden layers with 150 nodes each generated the best performance among all scenarios, the analysis was conducted on networks with this architecture.

**Figure 7:**
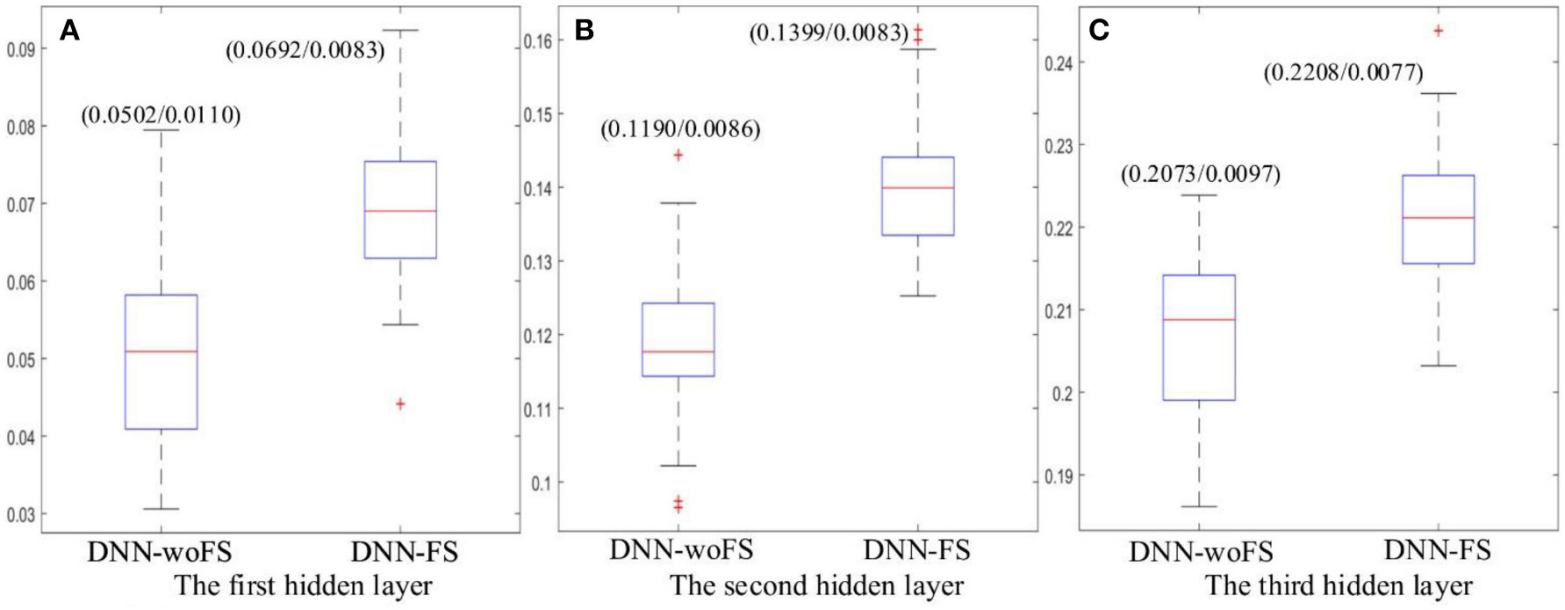
The mean Fisher's score and its *SD* (mean/*SD*) for each hidden layer from the DNN-FS and the DNN-woFS. **(A)** The first hidden layer. **(B)** The second hidden layer. **(C)** The third hidden layer.

The results showed that the higher layer always have higher mean Fisher's score in both DNN-FS and DNN-woFS (Figure 7). This demonstrates that the hierarchical property of the DNN can help it improve its discriminative power for classifying the two groups (ASD patients/TD controls) in a systematic manner, and multiple hidden layers are necessary. It was also observed that the DNN-FS has stronger discriminative power in each layer compared with DNN-woFS. Clearly, the proposed feature selection method improves the DNN classification accuracy by generating highly discriminative representations.

### Comparing the performance of the stacked SAEs in DNN-FS with other feature selection methods

**Figure 8:**
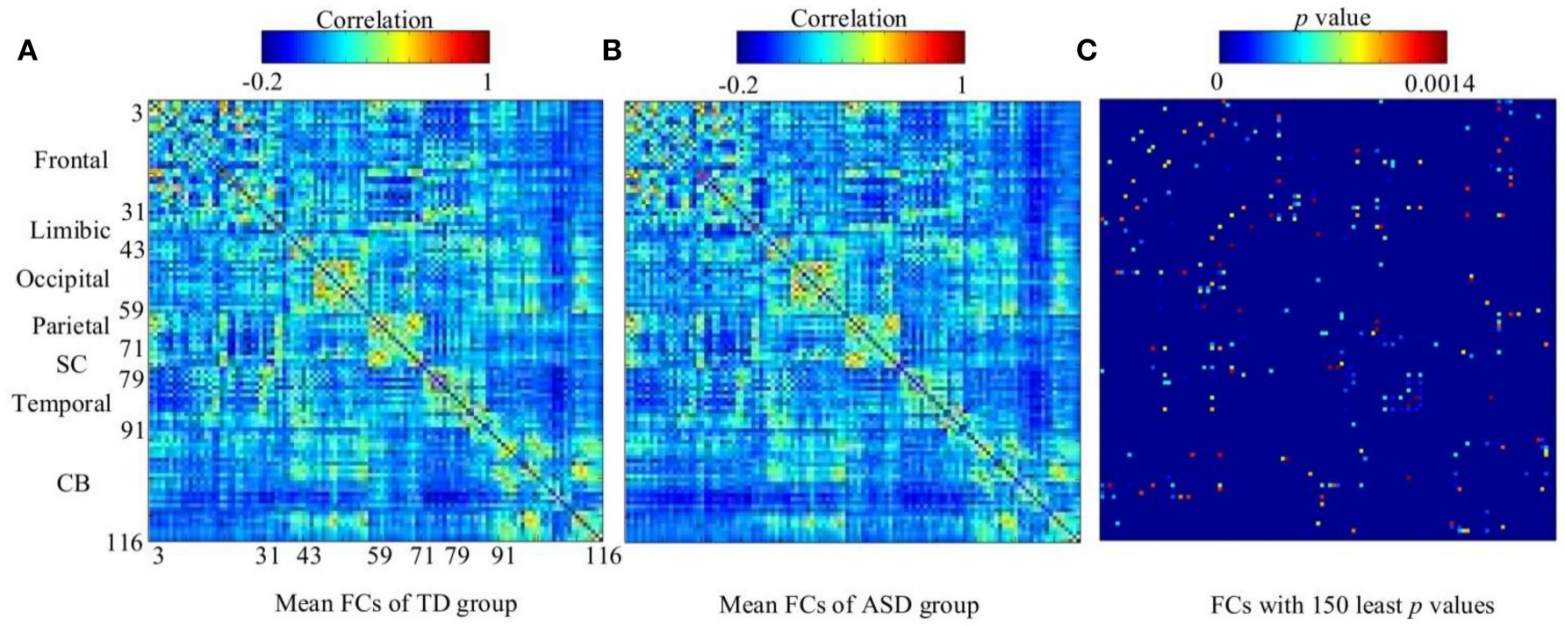
The visualization of group FCs. **(A)** Mean FCs of TD group (−0.2 ≤ *r* ≤ 1). **(B)** Mean FCs of ASD group (−0.2 ≤ *r* ≤ 1). **(C)** Group differences in the 150 FC patterns (evaluated by a two sample *t*-test with a threshold of *p* < 0.0014). Both x and y axes of each subfigure indicate areas in AAL atlas (SC, subcortical area, CB, cerebellum).

**Figure 9:**
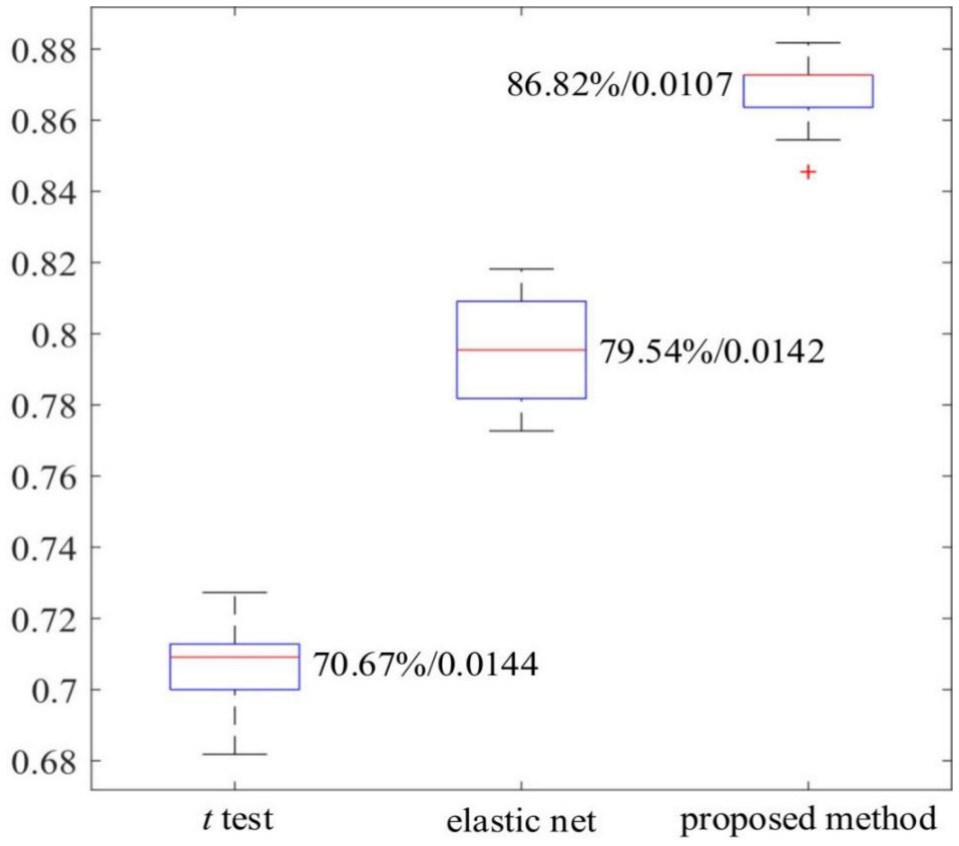
The comparison of three feature selection methods.

The work reported in this paper used stacked SAEs for dimension reduction in the DNN-FS framework. The proposed feature selection method helps the stacked SAEs generate reduced-dimension representations with high quality. Training with such representations, the classification accuracy of the SR model is improved significantly. To compare the data dimension reduction performance of the stacked SAEs in the DNN-FS with the performance of other methods, two other feature selection methods—two sample *t*-test and elastic net (Zou and Hastie, 2005)—were also implemented and tested, using individual FCs as the pool of features. The low-dimension representations generated by each method were used to train SR classifiers, and the performance of these classifiers as well as the DNN-FS-based SR classifier was evaluated on the test dataset. The training scheme for all three SR models was five-fold CV, and the training data was the same for them in each fold. As discussed above, the proposed feature selection method allowed a DNN with three layers and 150 nodes per hidden layer to generate the best performance, so this was the DNN architecture used here. In the two sample *t*-test, the distributions of each FC for the ASD and TD sets were compared, and the 150 FCs with the lowest *p*-values were selected out of 6,670 connections in each fold. The mean FCPs for the ASD and TD groups, and the 150 features with the lowest p-values are shown in Figure 8. The elastic net was also applied to select 150 FCs from the 6,670 FCs. To obtain reliable results, each method was run 10 times. In each trial, subjects were randomly divided into five-folds. The average accuracy as well as the standard deviation from each method is shown in Figure 9. As the figure illustrates, the DNN-FS method outperformed the other two methods, producing more robust, better quality low-dimensional representations for the SR model to classify TD controls.

### Visualization of significant FCs identified by learning

**Figure 10:**
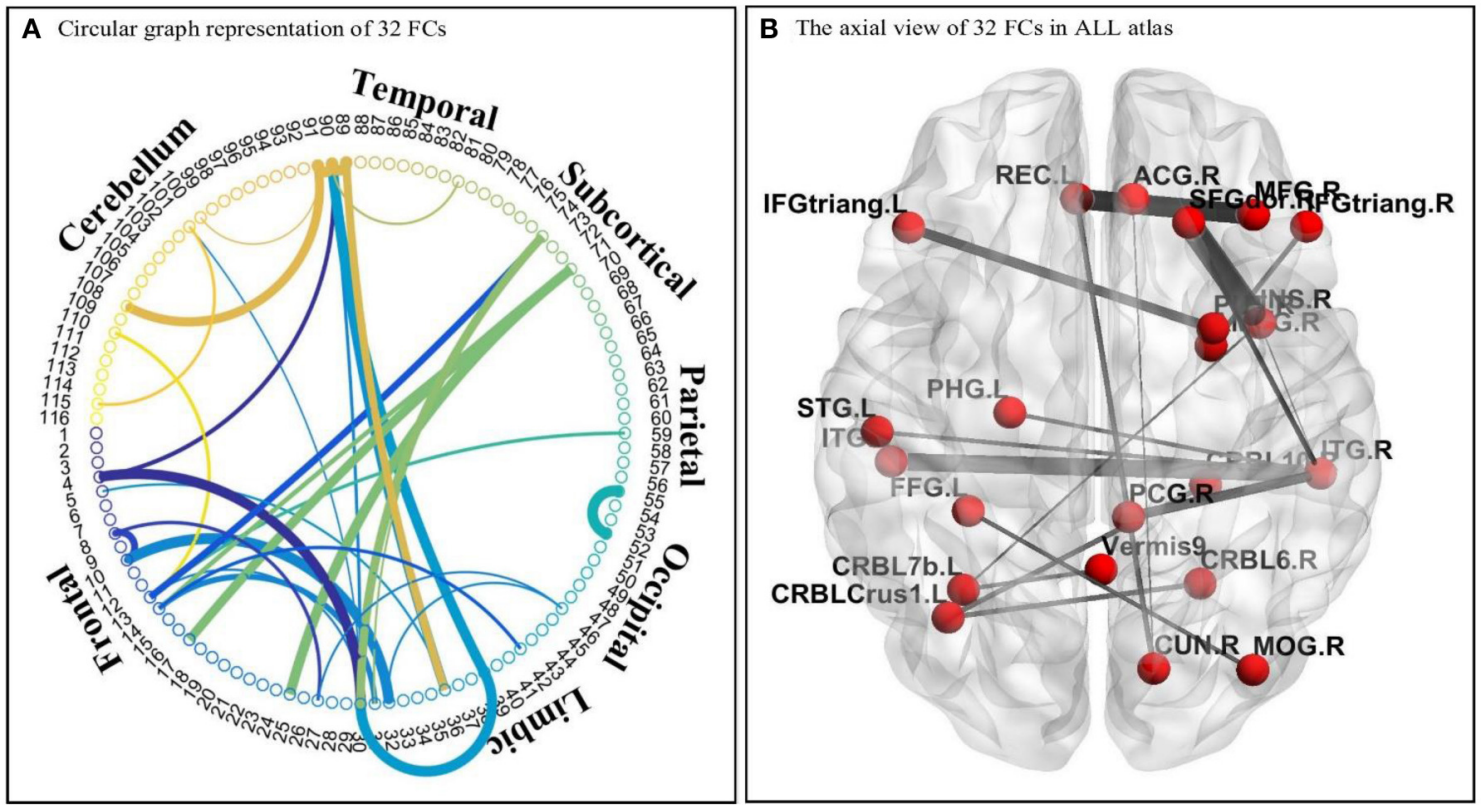
The visualization of 32 identified FC elements. **(A)** The circular visualization. Thicker connections indicate regions are strongly correlated, and vice versa. “circularGraph” toolbox (www.mathworks.com/matlabcentral) was applied to draw the figure. **(B)** The axial visualization in AAL atlas. Labels information was from AAL atlas. The thicker connection indicates two regions are strongly correlated, and vice versa. The BrainNet Viewer software (www.nitrc.org/projects/bnv) was applied to draw the figure.

As discussed earlier, the DNNs learn patterns from FCPs as features in a hierarchical manner: a feature in a higher layer is the linear combination of features from the layer below it. Thus, it is possible to look at what features each layer has learned, and use them to infer what elements of the FCPs were most important for classification and, therefore, for the diagnosis of ASD. Applying this approach, important FCs with significant discriminative power were identified, and visualized in the circular graph. More specifically, after conducting the experiment described in Section Analysis of Layer-Wise Discriminative Power, the four nodes with the largest Fisher's scores were located in the third hidden layer of the DNN-FS-based network with the best performance. For each of these, the two nodes with the largest connection weights from the second hidden layer were selected, giving up to eight second hidden layer nodes that contribute most to important features in the third hidden layer. The same process was iteratively applied on the first hidden layer and the input layer, resulting in 32 FCs which made high contribution to discriminate ASD patients from TD controls. These 32 FC elements are visualized in Figure 10. Furthermore, for purposes of network analysis, they are summarized in Table 6, with both terminal brain areas of each identified FC assigned to a brain functional network according to the literature. A few terminal areas that are not in any network defined in the extant research, i.e., the DM network, the FP network, the CO network and the CB network, are left blank. For each included FC, the mean *r* value for each group as well as the group difference was calculated, and the *p*-value obtained by the two sample *t*-test used to evaluate the significance. The significance threshold for discriminative power is *p* < 0.05. The FC was marked in green when its terminal brain regions were significantly positively correlated, and in red when they were significantly negatively correlated. All brain regions in the table were named by AAL labels (Tzourio-Mazoyer et al., 2002).

**Table 6:** The network analysis of 32 most significant FC elements.

**Connection ID**	**Regions**	**Network**	**Mean CC in ASD group**	**Mean CC in TDC group**	**Mean difference**	***P*-value**
1	(4) Frontal_Sup_R	CO	0.86	0.59	0.27	0.007
	(30) Insula_R	CO				
2	(74) putamen_R	CO	0.74	0.68	0.06	0.27
	(30) Insula_R	CO				
3	(31) Cingulum_Ant_L	CO	0.11	0.19	−0.08	0.33
	(74) Putamen_R	CO				
4	(36) Cingulum_Post_R	CO	0.56	0.68	−0.12	0.032
	(5) Frontal_Sup_Orb_L	CO				
5	(39) ParaHippocampal_L	DM	0.89	0.83	0.06	0.45
	(90) Temporal_Inf_R	DM				
6	(27) Rectus_L	DM	0.11	0.18	−0.07	0.23
	(46) Cuneus_R	DM				
7	(89) Temporal_Inf_L	DM	0.88	0.68	0.2	0.004
	(90) Parietal_Sup_L	DM				
8	(91) Cerebelum_Crus1_L	CB	0.04	0.11	−0.07	0.12
	(100) Cerebelum_6_R	CB				
9	(91) Cerebelum_Crus1_L	CB	0.76	0.83	−0.07	0.17
	(108) Cerebelum _10_R	CB				
10	(101) Creebelum_7b_L	CB	0.17	0.11	0.06	0.23
	(115) vermis_9	CB				
11	(32) Cingulum_Mid_L	FP	0.91	0.74	0.17	0.0043
	(10) Frontal _ Inf_Orb_L	FP				
12	(10) Frontal _ Inf_Orb_L	FP	0.56	0.55	0.01	0.56
	(8) Frontal_Mid_R	FP				
13	(59) Parietal_Sup_L	FP	0.23	0.19	0.04	0.78
	(13) Frontal_Inf_Frontal_Tri_L	FP				
14	(4) Frontal_Sup_R	CO	0.31	0.38	−0.07	0.015
	(90) Temporal_Inf_R	DM				
15	(36) Cingulum_Post_R	CO	0.78	0.66	0.12	0.002
	(90) Parietal_Sup_L	DM				
16	(32) Cingulum_Mid_L	FP	0.12	0.15	−0.03	0.19
	(46) Cuneus_R	DM				
17	(30) Insula_R	CO	0.07	0.11	−0.04	0.032
	(101) Cerebellum_7b_L	CB				
18	(8) Frontal_Mid_R	FP	0.23	0.46	−0.23	0.17
	(27) Rectus_L	DM				
19	(13) Frontal_Inf_Tri_L	FP	0.56	0.44	0.12	0.026
	(74) Putamen_R	CO				
20	(89) Temporal_Inf_L	DM	0.68	0.65	0.03	0.34
	(36) Cingulum_Post_R	CO				
21	(91) Cerebelum_Crus1_L	CB	0.88	0.75	0.13	0.045
	(90) Parietal_Sup_L	DM				
22	(110) Vermis_3	CB	0.23	0.18	0.05	0.34
	(13) Frontal_Inf_Tri_L	FP				
23	(39) ParaHippocampal_L	DM	0.89	0.84	0.05	0.036
	(30) Insula_R	CO				
24	(52) Occipital_Inf_L		0.91	0.85	0.06	0.45
	(55) Fusiform_L					
25	(14) Frontal_Inf_Tri_R	FP	0.23	0.16	0.07	0.76
	(42) Amygdala_L					
26	(71) Caudate_L		0.35	0.32	0.03	0.68
	(14) Frontal_Inf_Tri_R	FP				
27	(71) Caudate_L		0.78	0.83	−0.05	0.32
	(17) Rolandic_Oper_L					
28	(71) Caudate_L		0.87	0.84	0.09	0.61
	(25) Frontal_Med_Orb_L					
29	(81) Temporal_Sup_L	DM	0.12	0.18	−0.06	0.12
	(90) Temporal_Inf_R					
30	(30) Insula_R	CO	0.12	0.09	0.03	0.043
	(13) Frontal_Inf_Tri_L	FP				
31	(31) Cingulum_Ant_L	CO	0.45	0.58	−0.13	0.034
	(14) Frontal_Inf_Tri_R	FP				
32	(30) Insula_R	CO	0.2	0.23	−0.03	0.036
	(90) Parietal_Sup_L	DM				

*Red indicates the pair of regions are significantly negative correlated*.

*Green indicates the pair of regions are significantly positive correlated*.

## Discussion

In the present study, a DNN model with a novel feature selection method was developed for classifying ASD patients and TD controls based on the whole-brain FCPs. The first contribution of this work is the proposed feature selection method based on multiple trained SAEs to improve the quality of low-dimension representations learned from stacked SAEs. The proposed method (DNN-FS) was able to select diverse features with high discriminating power from a large feature pool consisting of all features from multiple trained SAEs, and these selected features, in turn, led to better classification of ASD patients and TD controls compared to the performance of DNN-woFS, and models with other feature selection methods (two sample *t*-test and elastic net). To test the efficacy of the method, both DNN-FS and DNN-woFS systems were trained and evaluated by the five-fold nested CV scheme under different comparison scenarios. Both models had the same architecture (the same number of hidden layers/nodes) in each scenario, and different scenarios used different architectures. The DNN-FS (3/150 hidden layers/nodes) obtained the best classification accuracy of **86.36%**. Most importantly, the DNN-FS outperformed the DNN-woFS for each comparison scenario. The most significant improvement, of **9.09%**, occurred when the architecture contained three hidden layers with 150 nodes each. Among many ASD diagnosis models, Deshpande et al. (2013) developed a recursive cluster elimination based support vector machine classifier using effective connectivity weights, behavior assessment scores, FC, and fractional anisotropy obtained from DTI data. The model achieved a maximum classification accuracy of 95.9%. Unlike the work presented here, they combined analysis of multiple types of data. The feature selection part of the model was thus able to extract and integrate discriminating features from heterogeneous data, which potentially enhanced classification accuracy. In addition, the number of ROIs (18) defined in their model was small, which kept their input data dimension a reasonable size compared to the sample size (30 adolescents and young adults), and inhibited overfitting. However, this method still needs to be validated on a larger dataset. Nielsen et al. (2013) developed a classification model to perform ASD classification, but only achieved up to 60% accuracy. They collected data from multiple sites of ABIDE I, and different sites had different imaging protocols and quality control protocols. Training the model with a large dataset was the most likely reason for obtaining the low accuracy. In addition, the lower heterogeneity of the large dataset might be another reason for the low accuracy. Compared to the dataset (964) they used, the present study used a smaller dataset from one site of ABIDE I, so the data quality is expected to be higher. To avoid overfitting of the DNN model, a nested cross validation evaluation scheme was applied. The effectiveness of the proposed feature selection method was also compared with others feature selection methods: two sample *t*-test and elastic net. Results showed that the classification model trained by representations from the stacked SAEs outperformed the models trained by features from other two methods. A comparison of the average Fisher's score of features in each layer in the DNN-FS and DNN-woFS also showed that discriminative power increased layer-wise in both DNN-FS and DNN-woFS, with higher layers providing more discrimination.

The second contribution of this work is a DNN-based biomarker identification method to locate 32 FCs associated with ASD, and exploration of the biological implications of the findings. Among these 32 FC elements, 13 were within pre-defined brain networks including CO, DM, CB and FP, and 19 were between-network FCs. 13 FCs were statistically significant (*p* < 0.05) between ASD and TD groups. Five significant FCs (1, 17, 23, 30, and 32 in Table 6) were associated with the insula. The previous study showed that this area was involved in interoceptive, affective, and empathic processes. Network analysis indicated that it was uniquely positioned as a hub mediating interactions between large-scale networks involved in externally- and internally-oriented cognitive processing, and it was a consistent locus of hypo- and hyper- activity in autism (Uddin and Menon, 2009). Menon and Uddin (2010) have hypothesized that impaired hub function of the anterior insula would reduce the ability of people with ASD to flexibly move from the executive control networks to the default mode. In the findings, one FC (1 in Table 6) was within-network (CO), and four FCs were between-network (one CO and CB, two DM and CO, one CO and CP). The results indicated that both hyper- and hypo-connectivities associated with the insula were existed within certain network or between networks, which can back up the point view in literature. Four significant FCs (4, 11, 15, and 31 in Table 6) were detected associated with the cingulate cortex. The right posterior cingulum were strongly correlated with the superior parietal gyrus, and were weakly correlated with the superior frontal gyrus in the ASD group. The left anterior cingulum was weakly correlated with the right pars triangularis, and the left middle cingulum was strongly correlated with the left pars orbitalis in the ASD group. The connections of the cingulate cortex to other brain structures are extensive, and thus the functions of the region are varied and complex. It makes important contributions to emotion, and various types of cognition such as the decision-making and the management of social behavior (Ikuta et al., 2014). The findings can help people to explore the neurological basis associated with abnormal symptoms in emotion and cognition. Meanwhile, another four ASD-related significant FCs were identified: LITG was strongly connected to LSPL (7 in Table 6), RSFG was weakly connected with RITG (14 in Table 6), LPT was strongly connected RP (19 in Table 6), and LCCB was strongly connected to LSPL (21 in Table 6). Brain areas associated with these FCs are involved in different brain functions such as spatial orientation (LSPL), visual shape processing (LITG&RITG), self-awareness (RSFG), and motor skills (LPT; Frackowiak, 2004; Lee et al., 2007). The dysfunctionality of certain brain areas might lead to such abnormal FCs in autistic children. However, the mechanism is unclear, and needed to be explored in the future. Other than above findings, 19 FCs which were not statistical significant were detected by the method too. They are not, respectively, discriminating may be because the sample size was not large enough to build the significant statistical power for each of them. However, they should be important in the classification task because all FCs made contribution during the learning process, and the model performance is supposed to be decreased if any of them is missing.

In the future, we are going to extend the current research in two aspects. First of all, the proposed method will be tested on datasets from cohorts in different age groups. Age is an important factor in the ASD diagnosis. ASD biomarkers may be altered due to the age difference. It is especially meaningful to make a convincing prediction before children develop ASD symptoms at their early age. Hazlett et al. (2017) demonstrated that they can make reasonably accurate forecast about which of these high-risk infants will later develop ASD themselves by examining the growth rate of brain volume of infants between 6 and 24 months based on anatomical brain images. The finding motivates us to evaluate the proposed method on a dataset from early-age children. NDAR (Hall et al., 2012) is an NIH-funded research data repository that aims to accelerate progress in ASD research, and it contains neuroimaging datasets from cohorts in different age groups. We plan to validate the generalization of the method on multiple NDAR datasets from infants, adolescents, and adults. We are optimistic about the model classification performance. No matter what the age group the dataset belongs to, the strength difference of certain FCs should exist between ASD patients and the age-matched TD controls. The DNN-FS can not only capture the discriminating FCs but also can learn high-quality discriminating features from the whole-brain FCP to enhance the classification accuracy of the SR model. However, the identified ASD-related FCs may be altered in different age groups. So it is necessary to work with neuroscientists closely to explore the neurological basis of identified FCs in each age groups.

Second of all, more systematic methods will be developed to decide the size of the feature pool for the proposed feature selection method, and to determine how the size affects the final performance. In addition, we will continue to explore the way of deciding the architecture (No. of layers/No. of nodes per layer) of the DNN-FS. It was found that the performance of the DNN-FS did not increase by adding more hidden layer nodes when the number of hidden layers was fixed. The most plausible explanation is that additional hidden layer nodes may learn many redundant features, which decreases the feature learning capacity of the stacked SAEs and further reduces classification accuracy. It was also found that the performance of the DNN-FS cannot be increased simply by adding more hidden layers when the number of hidden layer nodes is fixed. The most reasonable explanation is that the gradients vanish quickly in lower layers when the DNN has a very deep architecture, and even the pre-training step is not able to help much. Both these issues will be studied further as part of future research to obtain optimal DNN-FSs for classifying ASD patients and TD controls.

## Ethics statement

The Autism data used in this research was acquired through the public ABIDE I database through a data use agreement. The Autism database has de-identified all the patient health information (PHI) associated with the data. The original study to collect patients data was approved by IRB at University of Michigan. The potential risks of this project are limited to the unauthorized usage of the Autism data. To mitigate the potential risk, we will take the utmost care to ensure that there is no abuse of protected information.

## Author contributions

XG, LL, and AM conceived the project idea. XG implemented the method and performed the experiments. LL supervised the project. AM supervised the development of the feature selection method. KD and CE provided the detailed analysis of brain FCs discovered by the method. HL provided critical suggestions for the experiments design.

### Conflict of interest statement

The authors declare that the research was conducted in the absence of any commercial or financial relationships that could be construed as a potential conflict of interest.
